# The In Vitro Potential of 1-(1*H*-indol-3-yl) Derivatives against *Candida* spp. and *Aspergillus niger* as Tyrosinase Inhibitors

**DOI:** 10.3390/microorganisms9102070

**Published:** 2021-10-01

**Authors:** Teresa Gervasi, Giovanna Ginestra, Francesca Mancuso, Davide Barreca, Laura De Luca, Giuseppina Mandalari

**Affiliations:** 1 Department of Biomedical and Dental Sciences and Morphofunctional Imaging, University of Messina, 98125 Messina, Italy; teresa.gervasi@unime.it; 2 Department of Chemical, Biological, Pharmaceutical and Environmental Science, University of Messina, 98166 Messina, Italy; giovanna.ginestra@unime.it (G.G.); francesca.mancuso@unime.it (F.M.); davide.barreca@unime.it (D.B.)

**Keywords:** 1-(1*H*-indol-3-yl) derivatives, tyrosinase inhibitors, *Candida* spp., *Aspergillus niger*, antifungal

## Abstract

Given the increased antimicrobial resistance, global effort is currently focused on the identification of novel compounds, both of natural and chemical origin. The present study reports on the antifungal potential of 1-(1*H*-indol-3-yl) derivatives, previously known as tyrosinase inhibitors. The effect of seven compounds (indicated as **3a**–**g**) was determined against *Candida albicans* ATCC 10531, three clinical isolates of *Candida albicans*, two clinical isolates of *Candida glabrata*, two clinical isolates of *Candida parapsilosis* and *Aspergillus niger* ATCC 16404. The effect of these derivatives on tyrosinase enzymatic activity was also evaluated. Results showed a fungicidal activity of compounds **3b**, **3c** and **3e** against all tested strains at concentrations ranging between 0.250 and 1 mg/mL. Furthermore, the association between **3c** and fluconazole and between **3b** and caspofungin showed a trend of indifference tending toward synergism. Compound **3c** was also able to inhibit microbial tyrosinase up to ~28% at the concentration of 0.250 mg/mL. These data could help provide novel therapeutics for topical use to treat fungal infections and increase the potential effectiveness of the association between novel compounds and commercial antifungals in order to combat drug resistance.

## 1. Introduction

The current therapeutic drugs for *Candida* spp. infections are limited to five main classes of compounds, namely, polyenes, allylamines, azoles, fluoropyrimidines, and echinocandins, with amphotericin B, terbinafine, fluconazole, 5-fluorocytosine, and caspofungin as principal representatives [1]. The cell wall represents the main target for echinocandins and nikkomycins, whereas polyenes target the membrane phospholipid bilayer; azoles, allylamines and phenyl-morphololines block the synthesis of sterol, soldarin and flucytosine target the protein synthesis and nucleic acid synthesis, respectively, whereas griseofulvin acts on the microtubule assembly. However, given the increased incidence of *Candida* spp. community-based and hospital-acquired infections [2], more effort has recently focused on the identification of novel therapeutics. In immunocompetent subjects, *Candida* spp. are responsible for mucosal infections, including thrush and vaginitis, which could lead to invasive candidiasis in immunocompromised patients, especially with new species becoming multi-drug resistant [3]. Moreover, *C. albicans* could cause cerebritis and a mild memory impairment [4].

Tyrosinase inhibitors have been widely investigated, and their sources include plants and microbes, as well as semisynthetic and synthetic origins [5]. Tyrosinase is a multi-copper enzyme extensively distributed in different organisms, where it plays an important role in melanogenesis and enzymatic browning. Its role has also been studied to identify novel therapeutics preventing skin pigmentation and melanoma [6,7]. In addition to their antimycotic effect, azole inhibitors have been shown to have anti-inflammatory and anti-oxidant effects, as well as the potential to inhibit melanogenesis [8]. Typically, tyrosinase inhibitors present a monophenolic substrate, including tyrosine, or a diphenolic substrate, such as L-dopa, and activity is assessed based on dopachrome formation. Taslimi evaluated the anti-melanogenesis effect of several compounds obtained by natural sources, identifying natural phenols with inhibition against tyrosinase (IC50 values ranging between 2.37 and 7.90 µM) [9]. Cairone et al. have recently reported on the inhibitory activity towards acetylcholinesterase, butyrylcholinesterase, α-amylase, α-glucosidase and tyrosinase exerted by Clery strawberries, as well as their antifungal activity against *Candida albicans* [10].

The antifungal activity of an essential oil extracted from pistachio hulls against standard and clinical strains of *C. albicans*, *C. glabrata* and *C. parapsilopsis*, both alone and in combination with antifungal drugs, has been previously tested [11]. Furthermore, the 3-(4-benzylpiperidin-1-yl)-1-(1*H*-indol-3-yl)propan-1-one (**1a**) was identified as a promising mushroom tyrosinase inhibitor, and a number of new analogues were then synthesized [12,13]. The aim of the present work was to test a series of 1-(1*H*-indol-3-yl) derivatives against standard and clinical isolates of *Candida* spp. and *Aspergillus niger*. Their potential mechanisms of action as tyrosinase inhibitors were also evaluated.

## 2. Materials and Methods

### 2.1. Chemistry

All reagents were bought from common commercial suppliers and used without further purification. Microwave-assisted reactions were carried out in a Focused Microwave TM Synthesis System, Model Discover (CEM Technology Ltd. Buckingham, UK). Melting points were determined using Buchi B-545 apparatus (BUCHI Labortechnik AG Flawil, Switzerland) and were uncorrected. By combustion analysis (C, H, N) carried out on a Carlo Erba Model 1106-Elemental Analyser, the purity of synthesized compounds was determined; the results confirmed ≥ 95% purity. Merck Silica Gel 60 F254 plates were used for analytical TLC (Merck KGaA, Darmstadt, Germany). Flash Chromatography (FC) was carried out on a Biotage SP1 EXP (Biotage AB Uppsala, Sweden). ^1*H*^-NMR and ^13^C-NMR spectra were measured in dimethylsulfoxide-δ_6_ (DMSO-δ_6_) with a Varian Gemini 500 spectrometer (Varian Inc. Palo Alto, California USA); chemical shifts are expressed in δ (ppm) and coupling constants (*J*) in hertz. The spectral data for compounds **2a**–**b**, **2d**–**g** and **3a**–**3g** were consistent with those reported in the literature [12,13,14]. The ^13^C-NMR data for compound **3b** are reported as a representative of the entire compound set.

### 2.2. General Synthetic Procedure for 1-(1H-indol-3-yl)ethanone Derivatives (***2a**–**b*** and ***2d**–**g***)

The intermediates **2a**–**b** and **2d**–**g** were synthesized according to a previously reported experimental procedure [12,13]. Phosphorus oxychloride (POCl_3_) (1 molar equivalent) was added to dimethylacetamide (DMA) (3 molar equivalents) in an ice bath. Then, a suitable indole (**1a**–**1b**, **1d**–**g**) (0.1 molar equivalents) was added, and the reaction mixture was stirred at room temperature for 24 h, then poured and basified with a NaOH solution (4N). Furthermore, the mixture was extracted with EtOAc (3 x 10mL) and dried over Na_2_SO_4_. The solvent was removed under reduced pressure, and the crude was treated with a solution of diethyl ether (Et_2_O) and dichloromethane (CH_2_Cl_2_) to produce the desired 1-(1*H*-indol-3-yl) ethanone derivatives (**2a**–**b** and **2d**–**g**).

### 2.3. General Synthetic Procedure for Compounds ***3a**–**c**, **3di**, **3e**–**g***

To a stirred solution of the appropriate 1-(1*H*-indol-3-yl) ethanone derivatives **2a**–**2b** and **2d**–**g** (1 molar equivalent) in N,N-dimetilformamide (DMF) (3 mL), paraformaldehyde (1.3 molar equivalents) and 4-benzylpiperidine or 4-fluorobenzylpiperidine hydrochloride (1.1 molar equivalent) were added. Hydrochloric acid (37%) was used in catalytic amount. Then, the mixture was subjected to microwave irradiation at 250 W at 80 °C for 3 min. The progress of the reaction was monitored by thin layer chromatography (TLC) using a solution of CH_2_Cl_2_/CH_3_OH (9:1, *v*/*v*) as the eluent. The mixture was quenched with H_2_O (10 mL) and extracted with EtOAc (3 × 10 mL). The aqueous layer was alkalized with NaOH (2N) and extracted with EtOAc (3 × 10 mL). The combined organic layers were dried over Na_2_SO_4_ and concentrated in vacuo. The crude desired compound was purified by flash chromatography (CH_2_Cl_2_/CH_3_OH, 9:1, *v*/*v*) and crystallized by treatment with Et_2_O to afford compounds **3a**–**3c**, **3d** and **3e**–**g** as white powders.

^13^C-NMR data for 3-(4-benzyl-1-piperidyl)-1-(6-methoxy-1*H*-indol-3-yl)propan-1-one, **3b**.

Colourless solid. Yield: 42%. M.p.: 187–188 °C; ^13^C-NMR (126 MHz, DMSO-d_6_): 18.72, 27.23, 31.72, 37.02, 42.39, 50.47, 55.35, 55.54, 56.22, 95.32, 111.74, 116.06, 117.03, 119.39, 119.77, 125.86, 126.18, 128.23, 128.44, 127.07, 129.16, 137.75, 156.60, 191.60. Anal. calcd. for C_24_H_28_N_2_O_2_: C, 76.56; H, 7.50; N, 7.44. Found C, 76.59; H, 7.53; N, 7.47.

### 2.4. Synthetic Procedure for 3-(4-Benzylpiperidin-1-yl)-1-(6-hydroxy-1H-indol-3-yl) Propan-1-one (***3d***)

The precursor 3-(4-Benzylpiperidin-1-yl)-1-(6-methoxy-1*H*-indol-3-yl)propan-1-one (**3di**, 1 molar equivalent) was dissolved in CH_2_Cl_2_ (5 mL). Boron tribromide (BBr_3_, 1 M in DCM) (6 molar equivalents) was added to the mixture under a atmosphere, which was then stirred overnight at room temperature. Successively, the reaction crude was quenched with CH_3_OH (7 mL) in an ice bath and, therefore, the solvent was removed under reduced pressure. The solid residue was dissolved in EtOAc (10 mL), washed firstly with H_2_O (3 × 10 mL) and then with NaHCO_3_ saturated aqueous solution (2 × 10 mL). The organic layer was dried with Na_2_SO_4_ and evaporated in vacuo. The crude was purified by flash chromatography (CH_2_Cl_2_/CH_3_OH, 9:1, *v*/*v*) and recrystallized by treatment with EtOH and Et_2_O to afford the final compound as white powder (**3d**).

### 2.5. Microbial Strains and Culture Conditions

The following strains were used for the antifungal testing*: C. albicans* ATCC 10231, 3 clinical strains of *C. albicans* (12, 13, 16), 2 clinical strains of *C. parapsilosis* (30, 34), 2 clinical strains of *C. glabrata* (9, 33) and *Aspergillus niger* ATCC16404. All clinical *Candida* isolates were obtained at the IRCCS Centro Neurolesi “Bonino-Pulejo” hospital, Messina, Italy, and characterized as previously reported [11]. *Candida* strains were grown in RPMI 1640 (Sigma, Italy) at 30 °C for 24 h. For minimal fungicidal determination and killing curves, Sabouraud Dextrose Agar (Oxoid) was used. *Aspergillus niger* was grown in Sabouraud Dextrose Agar at 30 °C for 7 days as previously reported [15].

### 2.6. Susceptibility Studies

For the susceptibility studies, 1-(1*H*-indol-3-yl) derivatives (**3a**–**g**) were dissolved in DMSO at the concentration of 10 mg/mL. The minimum inhibitory concentration (MIC) and the minimum fungicidal concentration (MFC) of 1-(1*H*-indol-3-yl) derivatives and the antifungal compounds fluconazole and caspofungin (Sigma Aldrich, Italy) were determined by following the CLSI guidelines [16]. Serial dilutions were performed in the growth medium at concentrations between 1.000 and 0.391 mg/mL for the 1-(1*H*-indol-3-yl) derivatives, 64 and 0.0625 μg/mL for fluconazole, 2 and 0.00195 μg/mL for caspofungin. A positive control was included in each assay. Minimal fungicidal concentration (MFC) was determined by transferring each clear sample (20 μL) to an agar plate incubated at 30 °C for 48 h. The MFC was defined as the lowest extract concentration that killed 99.9% of the final inocula after 24–48 h incubation. The MIC was defined as the lowest concentration inhibiting the visible growth of the tested strains after incubation.

In order to test the efficacy of the combination of 1-(1*H*-indol-3-yl) derivatives and antifungal compounds, the ‘checkerboard’ procedure was followed [17]. MIC data for the 1-(1*H*-indol-3-yl) derivatives and each antifungal compound were converted into fractional inhibitory concentration (FIC), defined as the ratio of the concentration of the antimicrobial in an inhibitory concentration with a second compound to the concentration of the antimicrobial by itself, as follows:

FICI = MIC of A with B/MIC of A.

where A and B are the 1-(1*H*-indol-3-yl) derivative and the antifungal compound, respectively. All experiments were performed in triplicate on three independent days.

### 2.7. Erythrocytes Isolation and Haemolysis Assay

Blood was obtained by the venepuncture of healthy male volunteers and collected in heparinized tubes. Erythrocytes were separated from plasma and buffy coat and washed three times with l0 volumes of 0.9% NaCl and centrifuged at 2500 rpm for 5 min. During the last washing, the packed cells were resuspended in the incubation buffer (phosphate saline buffer), at pH 7.4 and utilized for subsequent experiments. The amounts of erythrocytes were selected to obtain, following dilution with distilled water, a value of Abs at 576 nm of 1.0. Erythrocytes were incubated for 24 h at 37 °C, both in the absence and in the presence of 1.000, 0.500 0.250, 0.125 and 0.061 mg/mL final concentrations of compounds **3b** and **3c**. At the end of incubation time, cells were centrifuged at 2500 rpm for 5 min. The supernatant was analysed to detect the release of haemoglobin following absorbance changes at 576 nm. The data are expressed as a percentage (%) of the maximum haemolysis, obtained in the sample without additive and diluted to distilled water to obtain the maximum available haemolysis.

### 2.8. Partial Purification of Tyrosinase, Total Protein Content and Enzymes Activity

The fungal strains were grown in the appropriate medium, as above described, at 37 °C and adjusted with sterile medium to result in approximately 5 × 10^6^ CFU/mL. Fungal strains were then collected by centrifugation at 10,000× *g* rpm for 10 min at 4 °C, washed three times and re-suspended in 0.5 mL of phosphate buffered saline (0.1 M, pH 6.8) with 1 mM phenylmethylsulfonyl fluoride (PMSF) and 1 mM ethylenedinitrilotetracetic acid (EDTA). Cell suspensions were broken with ultrasound in an ice bath for 5 min (550 W, working at 10 s intervals), and cell debris was removed by centrifugation at 10,000 rpm for 10 min at 4 °C. The enzyme, present in the supernatant, was partially purified using a 20 kDa cut-off dialyzer (20 kDa, 546-00051 Wako Chemicals, Richmond, VA, USA) against the same buffer.

The number of international units presented was analysed according to the methods of Naraoka et al. [18]. One international unit (IU) of enzyme activity was described as the amount of enzyme catalysing the transformation of 1 µmole of substrate to product per min at pH 6.8 at 25 °C, using the molar absorption coefficient of dopachrome (3600 M^−1^ cm^−1^). The obtained sample was collected, stored in an ice bath and used for total protein content and enzyme activity determinations. Total protein content was determined according to Bradford [19] at 595 nm, using bovine serum albumin as a standard.

Tyrosinase inhibition was tested according to the method of Masamoto et al. with minor modifications [20]. Briefly, aliquots (0.05 mL) of the tested compounds at various concentrations (to reach the final concentration in the mix of 0.250–0.0615 mg/mL) were mixed with 0.5 mL of L-DOPA solution (1.25 mM), 0.9 mL of phosphate buffered saline (0.02 M, pH 6.8) and preincubated at 25 °C for 10 min. Then, 0.05 mL of an aqueous solution of the partial purified tyrosinase (~200 U/mL) was added to the samples. The reaction mixture was immediately monitored for the formation of dopachrome by measuring the linear increase in absorbance (Abs) at 475 nm. The extent of inhibition by the addition of samples is expressed as the inhibition percentage (%) and calculated as follows:

Inhibition% = [(A − B)/A] × 100

where A represents the difference in the incubation time between 0.5 and 1.0 min in the in the sample, while B is the difference in the incubation time between 0.5 and 1.0 min in the blank. Kojic acid was employed as a reference inhibitor. The non-specific absorbance of the reagents was subtracted from one of the corresponding samples before performing the calculation inactivating the enzyme by heat treatment before adding it to the reaction mix.

### 2.9. Partial Purification of Tyrosinase, Total Protein Content and Enzyme Activity

Data are presented as means ± standard deviations (S.D.). Data were analysed by one-way analysis of variance (ANOVA). The significance of the difference from the respective controls for each experimental test condition was assayed by using Tukey’s test for each paired experiment. A *p* < 0.05 was regarded as indicating a significant difference.

## 3. Results

### 3.1. Chemistry

**Figure 1:**
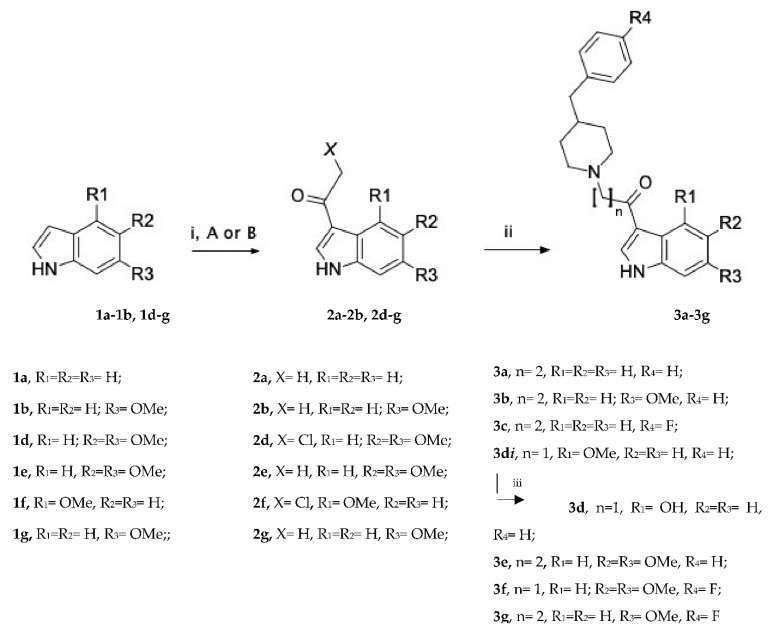
Reagents and conditions: (i) *A*, POCl_3_, DMA, rt, 24 h; *B,* ClCH_2_CON(CH_3_)_2_, POCl_3_, rt, 2.5 h; (ii) paraformaldehyde, 4-benzylpiperidine · HCl or 4-(4-fluorobenzyl)piperidine · HCl, HCl conc., DMF, MW, 80 °C, 3 min. (iii) BBr_3_, DCM, N_2_ atm., rt, overnight.

The synthesis of the desired compounds **3a**–**g** was performed by following a procedure previously reported [12,13], and it is summarized in Figure 1.

Specifically, the acetylation of the appropriate indole derivatives (**1a**–**b**, **1d**–**g**) was carried out by using N,N-dimethyl acetamide (DMA) or N,N-dimethylchloroacetamide (pathway i, A or B, respectively) and phosphorus oxychloride (POCl_3_), which afforded the corresponding intermediates **2a**–**b** and **2d**–**g**. The intermediates **2a**–**b**, **2e** and **2g** reacted with the suitable cycloalkylamines in the presence of paraformaldehyde and under microwave irradiation to produce the desired compounds **3a**–**3b**, **3e** and **3g** in high yields. Alternatively, the 2-chloro-1-(1*H*-indol-3-yl)ethan-1-one derivatives **2d** and **2f** were converted to the corresponding compounds **3di** and **3f** by coupling with the suitable benzylpiperidine derivatives. Finally, the demethylation of the methoxy group of the pioneer compound **3di** was performed using boron tribromide (BBr_3_) to yield the hydroxyindole derivative **3d**.

### 3.2. Antifungal Activity of 1-(1H-indol-3-yl) Derivatives

The MICs and MFCs values for the 1-(1*H*-indol-3-yl) derivatives (**3a**-**g**) are reported in Table 1 and Table 2, respectively. Results of negative controls using DMSO as a solvent indicated the complete absence of inhibition of all the strains tested (data not shown). All compounds, with the exception of **3a**, **3d** and **3f**, were active against the tested strains, with MIC values between 0.125 and 1.000 mg/mL. Compound **3f** was active against *A. niger* only. *C. parapsilosis* and *C. glabrata* strains were generally the most sensitive (complete inhibition achieved with a concentration of 0.125–0.250 mg/mL), followed by *C. albicans* strains (complete inhibition achieved with a concentration of 0.250–0.500 mg/mL). *A. niger* was overall more resistant compared to the yeast strains. Interestingly, the compound **3g** was only effective against one strain of *C. glabrata* (MIC values between 0.125 and 0.250 mg/mL) and one strain of *C. albicans* (MIC values between 0.250 and 0.500 mg/mL). The observed effect was fungicidal against all tested strains. The MIC and MFC values of the antifungal compounds fluconazole and caspofungin were obtained as previously reported [11].

Table 3 reports the FIC index calculated for the two most effective compounds (**3b** and **3c**) and each antifungal compound against *C. albicans* ATCC 10231, *C. albicans* strain 16, *C. glabrata* strain 33 and *C. parapsilosis* strain 34. Given that the FIC index interpretation depends on which definition is used, here, we report the value as synergistic if the FIC index is ≤0.5, additive or indifferent if >0.5 but ≤4 and antagonistic if >4 [21,22]. Indifference tending to synergism was observed between **3c** and floconazole against *C. albicans* strain 16 and *C. parapsilosis* strain 34 and between **3b** and caspofungin against *C. albicans* ATCC 20231 (FIC indices always <1). All the other combinations showed an indifferent effect, whereas no antagonistic interaction was observed against all tested strains.

### 3.3. Cytotoxicity Studies by Haemolytic Activity

**Figure 2:**
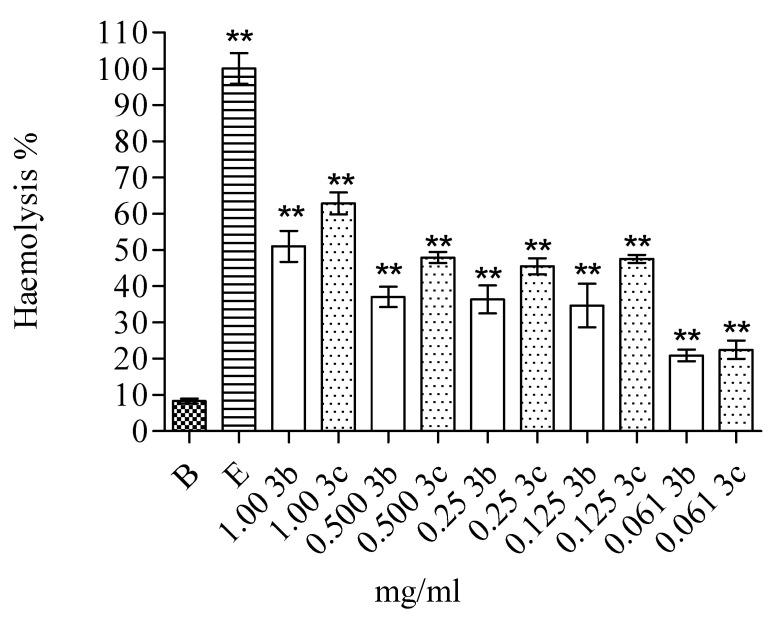
Haemolytic activity of compounds **3b** and **3c** and the concentration utilized in the experiments. The total haemolysis was obtained by diluting erythrocytes with distilled water (E). A control without additives to monitor spontaneous haemolysis was also performed (B). The data are expressed as means ± S.D. (*n* = 4). Asterisks (**) indicate a significant difference with respect to control (*p* < 0.05).

The cytotoxicity of the two most promising compounds was analysed by checking haemolytic activity against human red blood cells. The lysis percentage was evaluated by comparing the absorbances of each sample to the sample treated with distilled water to obtain the complete erythrocytes haemolysis (Figure 2). The positive control showed about 100% lysis (E), whereas the spontaneous haemolysis was ~7% (B). Compounds **3b** and **3c** were also analysed for potential haemolytic activity on erythrocytes. As reported in Figure 2, the incubation of erythrocytes with the tested compounds resulted in a haemolytic activity ranging from ~20% (for the lowest concentration tested) to ~60% (for the highest one) for both compounds.

### 3.4. Effect of 1-(1H-indol-3-yl) Derivatives on Tyrosinase Enzymatic Activity

**Figure 3:**
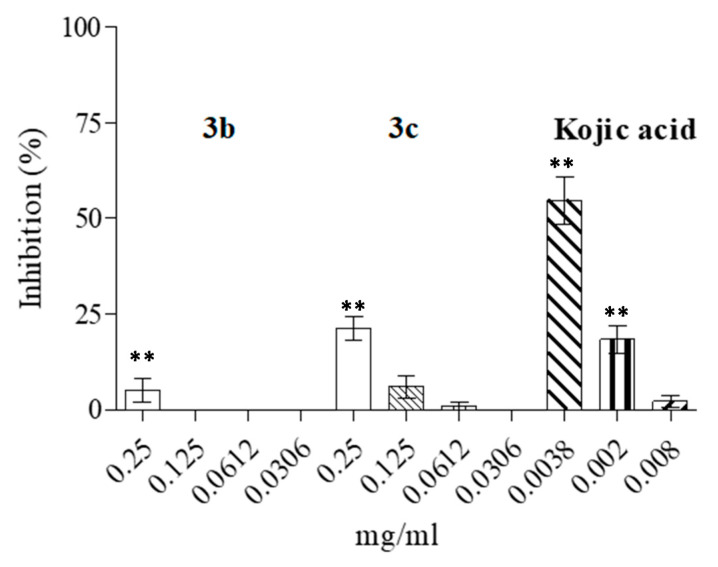
Inhibitory activity against partial purified tyrosinase obtained from *C. albicans* of the main compounds with antifungal activity and kojic acid (substrate:L-DOPA). Asterisks (**) indicate a significant difference with respect to control (*p* < 0.05).

In order to shed some light on the possible mechanisms of action of the compounds, we performed a partial purification of tyrosinase from the tested microorganisms and evaluated the inhibitory effects of the most promising compounds (**3b** and **3c**) at the concentrations of the experimental obtained MIC. The results showed an inhibition of the microorganism enzyme of up to about 28%, which may be, in part, responsible for the observed antimicrobial effects. As reported in Figure 3, compound **3c** was able to inhibit the enzyme up to ~28% at the maximum tested concentration of 0.250 mg/mL, whereas the inhibitory effects were clearly less evident at lower concentrations. On the other hand, compound **3b** was only able to perform a slight inhibition at the maximum tested concentration.

## 4. Discussion

The increase in antimicrobial resistance is considered a major health issue, and an estimated 700,000 people lose their life annually due to drug-resistant infections [23]. Therefore, global effort is currently focused on the development of novel therapeutics, both of natural and synthetic origin, to combat bacterial, fungal and viral resistance. Here, we have demonstrated that some 1-(1*H*-indol-3-yl) derivatives, known as tyrosinase inhibitors, have fungicidal activity against standard and clinical isolates of *Candida* spp. and the fungus *Aspergillus niger* and could potentially be used for topical formulations. Recently, a series of 2-aryl-3-azolyl-1-indolyl-propan-2-ol was synthetized and tested against *Candida albicans* and other *Candida* species: results showed a good antifungal potential of the compounds, with MIC values ranging between 0.005 and 1.25 μg/mL against *C. albicans* CA98001 [24]. The antimicrobial activity of novel indole derivatives containing 1,2,4-triazole, 1,3,4-thiadiazole and carbothioamide was evaluated against bacterial strains, including *Staphylococcus aureus*, methicillin-resistant *Staphylococcus aureus*, *Escherichia coli*, *Bacillus subtilis*, and the yeasts *Candida albicans* and *Candida krusei*: all compounds showed antimicrobial activity, with MIC values of 3.125–50 µg/mL [25]. Another study revealed that that 7-benzyloxyindole, 4-fluoroindole and 5-iodoindole effectively inhibited *C. albicans* biofilm formation compared to fluconazole [26].

Among tyrosinase inhibitors, a variety of different compounds, including polyphenols, benzaldehyde and benzoate derivatives, long-chain lipids and steroids, other natural (such as anthraquinones and dieckol) and synthetic (N-phenylthiourea, N-substituted-N-nitrosohydroxylamines, sildenafil, oxadiazole, oxazolones, and tetraketones types) inhibitors, as well as irreversible inactivators, have been identified [6]. Oliveira et al. [27] recently evaluated 16 analogues of coumaric and cinnamic acid as possible tyrosinase inhibitors; the results also indicated a growth inhibition in *Cryptococcus neoformans*, as well as an increase in amphotericin B antifungal activity (Coumaric acid analogues inhibit growth and melanin biosynthesis in *Cryptococcus neoformans* and potentialize amphotericin B antifungal activity).

A range of hydroxyl-substituted benzoic acid/cinnamic acid derivatives, whose antifungal potential has been widely investigated, have also been assessed for their tyrosinase inhibition and antimelanogenic activity [28]. The antifungal potential of two extracts obtained from millipede order Julida was recently evaluated against seven *Fusarium* species in combination with their acetylcholinesterase and tyrosinase inhibition activity [29].

Herein, we report the synthesis of derivatives **3a**–**3g** following a multistep process. All compounds were tested for antifungal activity against *Candida* spp. and *Aspergillus niger*. Among them, the best outcomes were obtained for **3b** and **3c**, which exhibited potent action against all the strains tested. Given the results obtained, it was found that the addition of substituents on the indole ring (e.g., compound **3b**) or benzyl tail (e.g., compound **3c**) play an important role in the modulation of antifungal activity. Indeed, moving from the unsubstituted derivative **3a** to the analogues bearing substituents on indole or benzyl portion, an excellent improvement of biological activity was observed. However, the simultaneous presence of substituents on the two heads of the molecule led to inactive compounds (e.g., **3f**). In addition, the analysis of the biological results pointed out the importance of the correct linker length between the indole ring and the benzylpiperidine moiety. Indeed, the two compounds, **3d** and **3f**, bearing a methylene bridge were found to be inactive against all the tested strains. The SAR information that emerged from this biological screening could be useful for further design aimed to identify optimizing antifungal candidates.

The use of novel antimicrobials in combination with traditional drugs could be useful to identify mechanisms of synergism and drug-resistant modulating properties. Here, we have shown that the association between compound **3c** and fluconazole was effective against *C. albicans* and *C. parapsilosis*. The same compound produced an inhibition of the microbial tyrosinase at the concentrations tested. The synergistic enhancements of antimicrobial activity could also be explored with magainins antimicrobial peptides, which may occur by indirect interactions through the lipid phase of bacterial membranes [30]. Recently, Vriens et al. [31] demonstrated that the radish defensins RsAFP1 and RsAFP2 act synergistically with caspofungin against the formation of *Candida albicans* biofilms).

## 5. Conclusions

In conclusion, we have demonstrated the fungicidal potential of 1-(1*H*-indol-3-yl) derivatives, which can potentially be used for topical formulations. In particular, the presence of the indole ring or benzyl tail on this present structure plays a crucial role in the demonstrated antifungal activity. Further studies are warranted to evaluate the mechanisms of action of potentially synergistic association to combat drug resistance as well as their effect on the partial inhibition of microbial tyrosinase.

## Figures and Tables

**Table 1:** MICs of 1-(1*H*-indol-3-yl) derivatives **3a**–**g** (expressed as mg/mL) against *Candida* spp. and *Aspergillus niger*.

STRAIN	3a	3b	3c	3d	3e	3f	3g
*Candida glabrata* strain *9*	>1.000	0.125–0.125	0.125–0.250	>1.000	0.250–0.250	>1.000	>1.000
*Candida glabrata* strain *33*	>1.000	0.125–0.125	0.125–0.125	>1.000	0.250–0.250	>1.000	0.125–0.250
*Candida parapsilosis* strain *30*	>1.000	0.125–0.125	0.125–0.125	>1.000	0.250–0.250	>1.000	>1.000
*Candida parapsilosis* strain *34*	>1.000	0.125–0.125	0.125–0.125	>1.000	0.250–0.250	>1.000	>1.000
*Candida albicans* strain *12*	>1.000	0.250–0.500	0.250–0.500	>1.000	0.500–1.000	>1.000	>1.000
*Candida albicans* strain *13*	>1.000	0.250–0.250	0.250–0.250	>1.000	0.500–1.000	>1.000	>1.000
*Candida albicans* strain *16*	>1.000	0.125–0.125	0.125–0.250	>1.000	0.500–1.000	>1.000	0.250–0.500
*Candida albicans ATCC* *10231*	>1.000	0.250–0.250	0.250–0.250	>1.000	0.500–1.000	>1.000	>1.000
*Aspergillus niger ATCC* *16404*	>1.000	0.500–0.500	0.500–0.500	>1.000	1.000–1.000	0.500–0.500	>1.000

MIC, minimum inhibitory concentration.

**Table 2:** MFCs of 1-(1*H*-indol-3-yl) derivatives **3a**–**g** (expressed as mg/mL) against *Candida* spp. and *Aspergillus niger*.

STRAIN	3a	3b	3c	3d	3e	3f	3g
*Candida glabrata* strain *9*	>1.000	0.250	0.250	>1.000	0.500	>1.000	>1.000
*Candida glabrata* strain *33*	>1.000	0.250	0.125	>1.000	0.500	>1.000	>1.000
*Candida parapsilosis* strain *30*	>1.000	0.250	0.125	>1.000	0.500	>1.000	>1.000
*Candida parapsilosis* strain *34*	>1.000	0.125	0.125	>1.000	0.500	>1.000	>1.000
*Candida albicans* strain *12*	>1.000	1.000	0.500	>1.000	0.500	>1.000	>1.000
*Candida albicans* strain *13*	>1.000	1.000	0.500	>1.000	0.500	>1.000	>1.000
*Candida albicans* strain *16*	>1.000	0.250	0.250	>1.000	0.500	>1.000	>1.000
*Candida albicans ATCC* *10231*	>1.000	0.500	0.250	>1.000	0.500	>1.000	>1.000
*Aspergillus niger ATCC* *16404*	>1.000	1.000	1.000	>1.000	1.000	>1.000	>1.000

MFC, minimum fungicidal concentration.

**Table 3:** FIC index of the association between **3b** and **3c** with reference antifungal (caspofungin and fluconazole) against ATCC and clinical *Candida* spp.

STRAIN	3b/Caspofungin	3b/Fluconazole	3c/Caspofungin	3c/Fluconazole
*Candida glabrata* strain *33*	1.50	1.06	2.00	2.00
*Candida parapsilosis* strain *34*	1.50	1.06	1.06	0.61
*Candida albicans* strain *16*	1.47	2.62	1.21	0.62
*Candida albicans ATCC* *10231*	0.75	3.00	1.25	1.10

## Data Availability

The data presented in this study are available on request from the corresponding author.
